# Supplementary material for: Spatial coherence of light inside three-dimensional media
Source: Nat Commun. 2021 Jul 7;12:4199. doi: 10.1038/s41467-021-23978-0 (PMC8263759; doi:10.1038/s41467-021-23978-0)
Supplement: Supplementary file 1 — Supplementary Information [file 41467_2021_23978_MOESM1_ESM.pdf]

# Spatial coherence of light inside three dimensional media: Supplementary information

Marco Leonetti,[1,2] Lorenzo Pattelli,[3,4] Simone De Panfilis,[1] Diederik S. Wiersma,[3,4,5] and Giancarlo Ruocco[1,6]

[1]Center for Life Nano science @ Sapienza, Isituto Italiano di Tecnologia, 00161 Roma, Italy
[2]Institute of Nanotechnology, CNR-NANOTEC, Rome, Italy
[3]Istituto Nazionale di Ricerca Metrologica (INRiM), 10135 Torino, Italy
[4]European Laboratory for Non-linear Spectroscopy (LENS), 50019 Sesto Fiorentino, Italy
[5]Department of Physics, Università di Firenze, 50019 Sesto Fiorentino (FI), Italy
[6]Dipartimento di Fisica, Universitá "La Sapienza", 00185 Roma, Italy

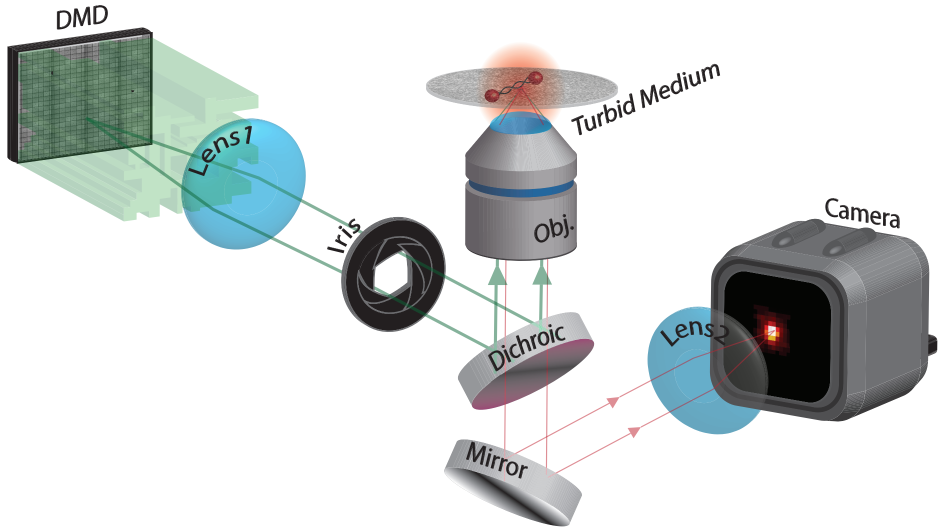


Supplementary Fig. 1. **Sketch of the experimental setup.** Light generated by a low noise stabilized, single mode laser (Azure Light, 0.5 W, 532 nm) impinges on a computer-controlled Digital Micromirror Device (DMD, Vialux V-7000, 1024×768 pixels, 13.7 µm pitch), and is directed by a two-lens system to the sample. An iris is used to select a specific area of the Fourier space of the DMD and avoid inhomogeneous contributions form high-intensity diffraction orders. The DMD is modulated with a random mask, in which micromirrors can take the "on" or the "off" position over a 100×100 pixels active area and imaged onto the sample. Light from a single Gatta-sted (their number density is tuned in order to have at most one single nanoruler in the field of view) is collected though a dichroic mirror and imaged on a single-photon camera (Evolve Delta 512 Camera). An $x$-$y$-$z$ piezometric actuator (Thorlabs MAX311D/M 3-Axis NanoMax Stage, Differential Drives, Closed-Loop Piezos) ensures the steady positioning of the sample.

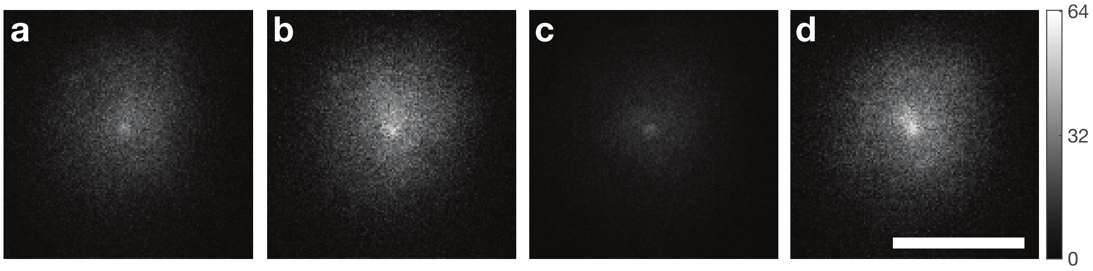


Supplementary Fig. 2. **Scattered fluorescence from buried nanoruler.** Image of a single nanoruler (with $D$ = 160 nm), buried in the scattering sample composed of ZnO in Agarose gel. The scale bar is 5 µm, the exposure time has been set to 200 ms. The different panels show the emission obtained for four different illumination patterns.

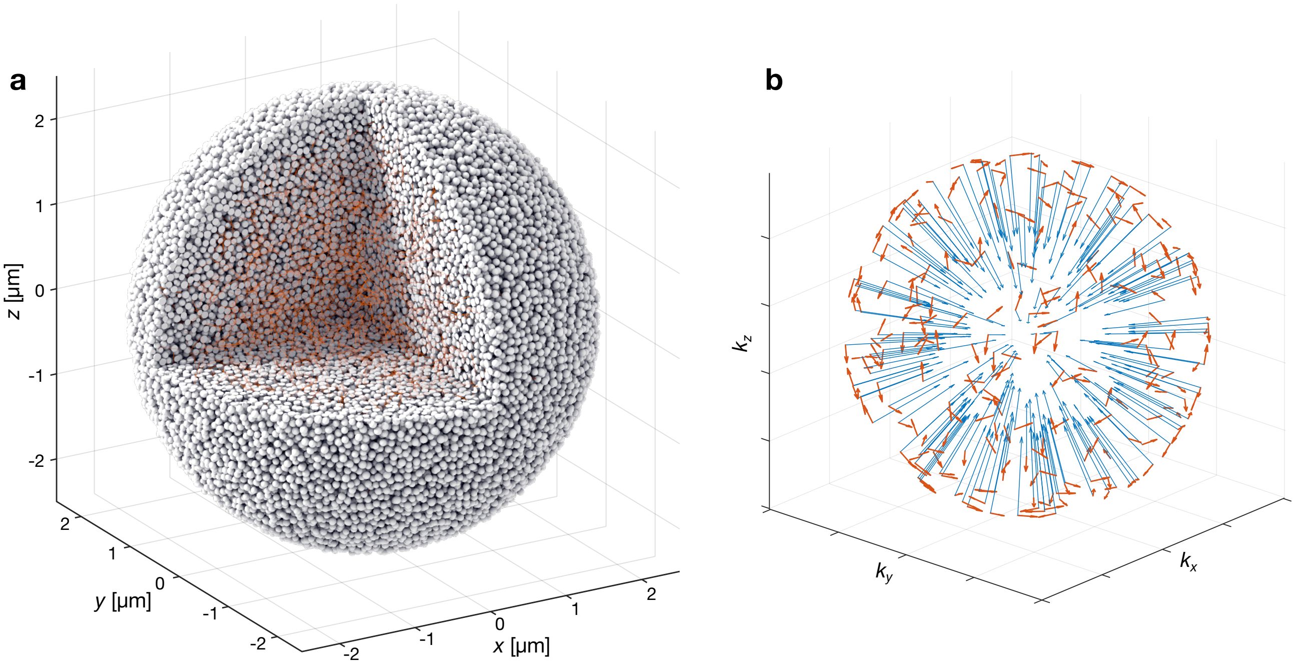


Supplementary Fig. 3. **Configuration used for rigorous calculations.** a) Spherical aggregate of particles used for generalized multiparticle Mie theory (GMMT) calculations. The sample comprises roughly $1.2 \times 10^5$ polydisperse spherical nanoparticles distributed with a ∼38 % volume fraction over a spherical domain with a diameter of 5 μm. A portion of the nanoparticle aggregate is removed to show the internal distribution of $10^4$ Gatta-sted (red, shaded), which are modeled as rigid segments with no transverse width. Gatta-sted are distributed uniformly with random orientation inside the aggregate so that they do not overlap with any particle and both extremities are well within the aggregate. b) Sample of 250 randomly rotated propagation and polarization unit vectors used to generate isotropic illumination conditions inside the bulk diffusive medium.

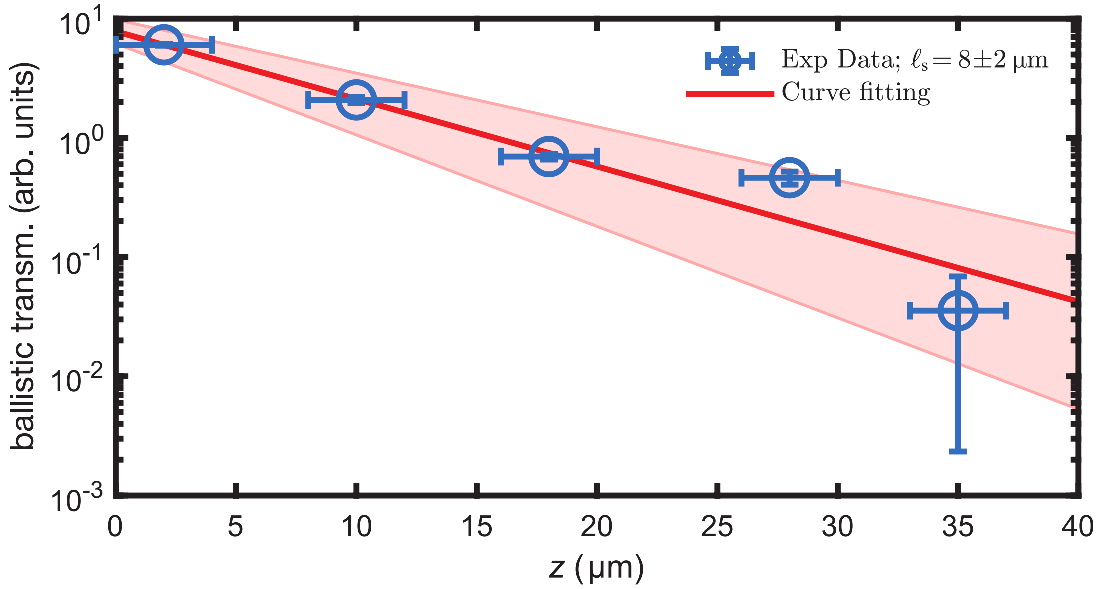


Supplementary Fig. 4. **Scattering mean free path estimation.** Dependence of the measured ballistic transmittance through ZnO-Agarose samples as a function of sample thickness. Experimental data are obtained using an Olympus FV1200MPE confocal microscope in the transmittance configuration (collection NA 0.04). Sample thickness is estimated by focusing on the glass substrate and sample surface while recording the piezo stage travel range. The error on the abscissa comes form the uncertainty on the correct identification of the focus plane (±2 μm). Error on the transmittance results from the statistics collected over 5 areas with a side of 2.5 μm each. The scattering mean free path is finally obtained via Lambert-Beer law fit, returning: $\ell_s = (8 \pm 2)$ μm. The shaded area is a guide to the eyes bounded between $\ell_s = 6$ μm and $\ell_s = 10$ μm

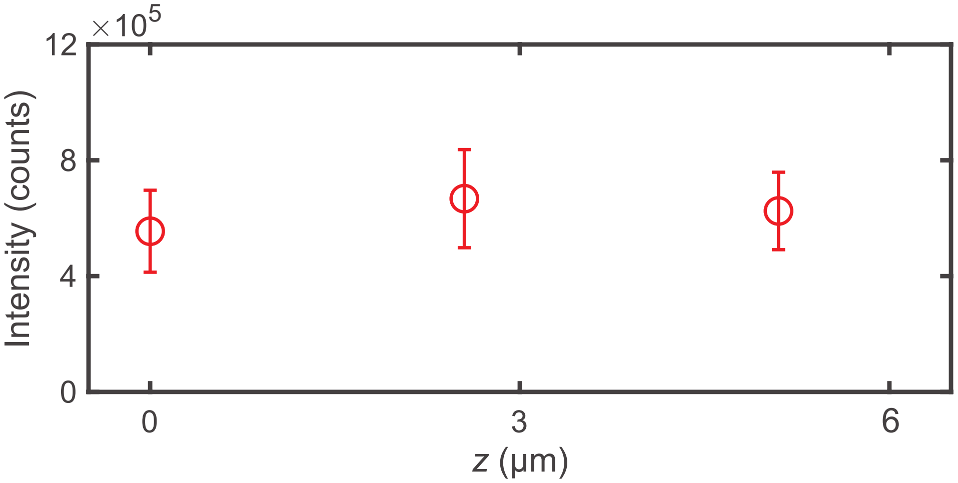


Supplementary Fig. 5. **Independence of fluorescence intensity on the distance from hydrophobic surfaces.** Measurement of the nanorulers emission intensity versus the distance $z$ from a glass slide covered with an hydrophobic PDMS layer. Error bars are obtained from the statistics over eight Gattaquant nanoruler per each $z$ value. The sample consists of nanorulers ($D$ = 160 nm Gattaquant) dispersed in a water-agarose gel matrix over a hydrophobic PDMS layer. Measurements have been performed with an Olympus FV1200MPE confocal microscope in reflectance configuration and with $z$-resolution 150 nm (smaller than marker size in the graph). Individual nanoruler emission intensity has been extracted by integrating counts in a 180 nm square region of interest at the $z$ value for which the nanoruler is in focus. No effects due to the proximity to a hydrophobic surface is noticeable from this measurement, as expected for a high quantum yield emitter.

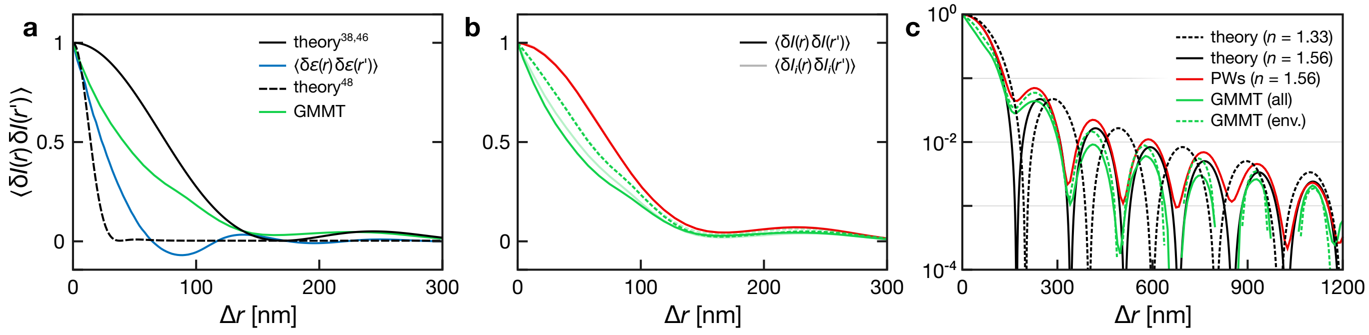


Supplementary Fig. 6. **Contributions to the width of intensity correlations**. (a) Comparison of numerically obtained intensity correlations using generalized multiparticle Mie theory (GMMT; green, solid) against the far-field prediction (black, solid) and a non-universal model (black, dashed) obtained from the width of the permittivity correlations (blue, solid). (b) Comparison of numerical intensity correlations obtained accounting for the presence of cross-polarization correlations (solid curves) or ignoring them (shaded curves), using both the GMMT model and a simple sum of randomly oriented plane waves (PWs). Of the 3 curve pairs shown in this plot, only that calculated over the whole scattering medium shows a visible difference. (c) Semi-log scale comparison of calculated $C_I$ curves against far field models obtained for $\lambda_0/1.56$ and $\lambda_0/1.33$.
